# The Potential Role of the Intestinal Micromilieu and Individual Microbes in the Immunobiology of Chimeric Antigen Receptor T-Cell Therapy

**DOI:** 10.3389/fimmu.2021.670286

**Published:** 2021-05-31

**Authors:** Maria-Luisa Schubert, Roman Rohrbach, Michael Schmitt, Christoph K. Stein-Thoeringer

**Affiliations:** ^1^ Klinik fuer Haematologie, Onkologie und Rheumatologie, Universitätsklinikum Heidelberg, Heidelberg, Germany; ^2^ Research Division Microbiome and Cancer, Deutsches Krebsforschungszentrum (DKFZ), Heidelberg, Germany; ^3^ Klinik fuer Medizinische Onkologie, Nationales Centrum für Tumorerkrankungen (NCT), Heidelberg, Germany

**Keywords:** microbiome, immunotherapy, antibiotic, CAR (chimeric antigen receptor) T cells, microbe-host association

## Abstract

Cellular immunotherapy with chimeric antigen receptor (CAR)-T cells (CARTs) represents a breakthrough in the treatment of hematologic malignancies. CARTs are genetically engineered hybrid receptors that combine antigen-specificity of monoclonal antibodies with T cell function to direct patient-derived T cells to kill malignant cells expressing the target (tumor) antigen. CARTs have been introduced into clinical medicine as CD19-targeted CARTs for refractory and relapsed B cell malignancies. Despite high initial response rates, current CART therapies are limited by a long-term loss of antitumor efficacy, the occurrence of toxicities, and the lack of biomarkers for predicting therapy and toxicity outcomes. In the past decade, the gut microbiome of mammals has been extensively studied and evidence is accumulating that human health, apart from our own genome, largely depends on microbes that are living in and on the human body. The microbiome encompasses more than 1000 bacterial species who collectively encode a metagenome that guides multifaceted, bidirectional host-microbiome interactions, primarily through the action of microbial metabolites. Increasing knowledge has been accumulated on the role of the gut microbiome in T cell-driven anticancer immunotherapy. It has been shown that antibiotics, dietary components and gut microbes reciprocally affect the efficacy and toxicity of allogeneic hematopoietic cell transplantation (allo HCT) as the prototype of T cell-based immunotherapy for hematologic malignancies, and that microbiome diversity metrics can predict clinical outcomes of allo HCTs. In this review, we will provide a comprehensive overview of the principles of CD19-CART immunotherapy and major aspects of the gut microbiome and its modulators that impact antitumor T cell transfer therapies. We will outline i) the extrinsic and intrinsic variables that can contribute to the complex interaction of the gut microbiome and host in CART immunotherapy, including ii) antibiotic administration affecting loss of colonization resistance, expansion of pathobionts and disturbed mucosal and immunological homeostasis, and ii) the role of specific gut commensals and their microbial virulence factors in host immunity and inflammation. Although the role of the gut microbiome in CART immunotherapy has only been marginally explored so far, this review may open a new chapter and views on putative connections and mechanisms.

## CAR-T Cell Therapy Transforms Immunotherapy Against Hematologic Malignancies

Primary cancer treatment relied for decades almost exclusively on surgery, chemotherapy and radiation therapy. With the development of monoclonal antibodies and the advent of stem cell transplantation, immunotherapy became a clinical option for the treatment of malignant diseases. Agents that induce or enhance antitumor responses, i.e. immune checkpoint inhibition (ICI) and adoptive T cell (ATC) therapy have revolutionized immunotherapeutic approaches. ICI using monoclonal antibodies against the cytotoxic T lymphocyte associated protein 4 (CTLA-4), the programmed cell death protein 1 (PD-1) or the programmed cell death ligand 1 (PD-1 L) (1) are now considered the standard of care in numerous solid and hematologic malignancies including advanced-stage melanoma, non-small-cell lung cancer (NSCLC), head and neck cancer, bladder cancer, or renal cell carcinoma (2). ATC therapy including tumor-infiltrating lymphocytes (TILs), T cell receptor (TCR)-engineered T cells and chimeric antigen receptor (CAR) gene-transduced T cells (CARTs) redirects T cells to tumor antigens. Therapy with TILs has achieved promising therapeutic results in melanoma (3–6) and TCR T cell receptor therapy is under clinical evaluation for different malignancies (NCT03686124, NCT03970382, NCT03691376). TILs and TCR T cell therapy are restricted to Human Leukocyte Antigen (HLA)-expressing antigens. In contrast, CARTs act in an HLA-independent manner and have the potential to effectively recognize target surface antigens, thus restricting immune evasion of malignant cells by HLA-downregulation.

CARTs constitute synthetic receptors composed of 1. an extracellular antigen-specific domain derived from an antibody’s single chain variable fragment (scFv), 2. a hinge and transmembrane segment, and 3. an intracellular domain that mediates activation and co-stimulation of the T cell that has been genetically engineered to express the CAR on the surface. Hence, CARTs combine the antigen-binding properties of antibodies with the effector functions of T cells. The intracellular CAR-domain defines different CART generations. First-generation CARs contain only the tyrosine-based ζ-signal-transducing subunit from the TCR/CD3 receptor complex and have displayed limited *in vivo* expansion and persistence of transduced T cells (7). Second-generation CARTs carry a costimulatory domain, e.g. CD28, 4-1BB (CD137), DAP-12 (8), OX40 (CD134) (9) or inducible T cell co-stimulator (ICOS) (10), adjacent to the TCR/CD3ζ-domain to mediate superior CART activation, proliferation and *in vivo* persistence (11). When directed against CD19, second-generation CARTs have demonstrated unprecedented clinical responses in a variety of relapsed and/or refractory (r/r) B cell malignancies including pediatric (12, 13) and adult (14, 15) acute lymphoblastic leukemia (ALL), chronic lymphocytic leukemia (CLL) (16–19), and other non-Hodgkin’s lymphoma (NHL) (20, 21).

Second-generation CARTs evaluated in the ZUMA-1 (diffuse large B-cell lymphoma (DLBCL) and primary mediastinal B-cell lymphoma (PMBCL)) (22, 23), ELIANA (ALL) (13) and JULIET (DLBCL) (24) as well as the ZUMA-2 trial (mantle cell lymphoma (MCL)) (21), achieved clinical responses in up to 93% of ALL (13, 25–27) and 82% (ZUMA-1) (22), 52% (JULIET) (28) and 92% (21) of NHL patients. Based on this pivotal trials, commercial CARTs axicabtagene ciloleucel (axi-cel) (29), tisagenlecleucel (tisa-cel) (30) and brexucabtagene autoleucel (brexu-cel) (31) were approved. These CARTs have become an integral part of the clinical hematologic practice within the authorized indications and have demonstrated efficacy also in the real-world setting (32–35).

Third-generation CARTs include two co-stimulatory molecules within their CAR constructs and have shown enhanced T cell activation *in vitro* and *in vivo*, sustained proliferation and tumor-lytic activity as well as reduced activation-induced cell death (36–39). Nonetheless, clinical evaluation is ongoing to assess if third-generation CARTs are advantageous in the clinical setting with regards to efficacy and safety (40, 41). Fourth-generation CARTs, i.e. engineered T cells redirected for universal cytokine killing (TRUCKs), endow additional modifications such as additional co-stimulatory ligands or cytokines to enhance their efficacy recruiting other effectors of the immune system (42, 43).

Besides CD19 as target, CARTs are under development against other tumor antigens, e. g. CD22 for ALL (44–46), CD30 for Hodgkin lymphoma and anaplastic large cell lymphoma (ALCL) (47, 48) or CD5 for T cell lymphoma (49). For multiple myeloma (MM), CARTs targeting the B cell maturation antigen (BCMA) (50–53) and for acute myeloid leukemia (AML) CARTs targeting CD33 (54), CD123 (55, 56) or CLL-1 (57) are under evaluation. Non-hematologic malignancies addressed by CARTs include glioblastoma (58–60) or neuroblastoma (61–63). CARTs to treat non-malignant indications, i.e. autoimmune (64) or infectious diseases, e. g. human immunodeficiency virus (HIV) infections (NCT0361719; NCT03240328) are also being clinically evaluated. Besides of T cells as sources for CART production, natural killer (NK) cells are broadening the application of CAR cells beyond the autologous T cell setting (65). Currently, 861 CAR trials are ongoing (clinicaltrials.gov; search for CAR cells; February 14^th^ 2021).

## T Cell Activation, Toxicity and Antitumor Action as Major Elements in the Pharmacobiology of CARTs

Upon encounter and binding of the CAR with the target antigen, CARTs get activated. Activation results in cytotoxicity towards the targeted cell and in immune activation by recruitment of other T cells and bystander immune cells. Depletion of recipient lymphocytes before CART administration enhances engraftment, persistence, and efficacy of CARTs due to the reduction of resident lymphocytes and the reduction of regulatory T cells (66). Furthermore, lymphodepletion has been shown to stimulate stromal cells to produce the cytokines IL-7 and IL-15, both associated with enhanced expansion of CARTs (67–69).

As cellular products, CARTs do not exhibit typical pharmacokinetic properties of traditional drugs and the unique biology of CARTs explains the specific toxicities associated with this therapy including cytokine release syndrome (CRS), neurotoxicity, cytopenia, on-target-off tumor effects (i.e., B cell aplasia and consecutive hypogammaglobulinemia in CD19 CART therapy) and infections.

CRS is frequently observed after CART treatment with CD19-specific CARTs (12, 13, 22) but also with CARTs targeting BCMA (50) and with other T-cell engaging therapies (70, 71). CRS is triggered by inflammatory mediators released directly by the CART and the activated bystander immune cell and results in a supraphysiologic inflammatory state (72–74). CRS manifests with constitutional symptoms such as fever associated with fatigue, myalgia, arthralgia, rigors or anorexia, but can progress to hypotension, tachycardia, tachypnea and hypoxia, arrhythmia, capillary-leak, coagulopathy, respiratory failure, shock and organ dysfunction (22, 75). The treatment of CRS involves symptomatic treatment as well as anti-cytokine treatment with anti-IL-6 antibodies and corticosteroids (28, 76, 77).

Neurotoxicity, referred to as immune effector cell-associated neurotoxicity syndrome (ICANS), is another toxicity commonly observed after CART treatment. ICANS typically presents with impairment of attention and confusion (26, 78–80) and can progress to depressed level of consciousness, coma, seizures, motor weakness, and cerebral edema (34, 81). Trafficking of CARTs, passive diffusion of cytokines into the central nervous system (CNS), endothelial activation with subsequent disruption of the blood-brain barrier and microglial and/or myeloid cell activation in the CNS have been suggested as underlying the pathophysiology of ICANs (14, 25, 80–83). For isolated ICANS, steroids are the first-line of therapy (76).

High-grade and long-term cytopenias are frequently observed after CART therapy and prone patients to infectious complications (13, 22, 23, 25, 28, 84–86). Also, B cell aplasia with secondary hypogammaglobulinemia due to the effects of CD19-directed CARTs on normal B cells can be associated with an increased risk of infections (87, 88).

## Infections and Antibiotic Treatments in CART Cell Patients

Besides cytopenia and hypogammaglobulinemia, further risk factors for infections in CART patients include the number of prior chemotherapeutic treatment regimens, impaired performance status at immunotherapy start, ALL as underlying disease, a reduced absolute neutrophil count at baseline, a high dose of administered CARTs and the use of corticosteroid treatment for management of CART toxicities (15, 73, 89). In fact, early and late infectious complications, primarily of bacterial and viral origin, after CART administration are common (32, 85, 90–92). Besides, invasive fungal infections (90, 93) and reactivation of latent DNA viruses are observed after CART treatment (79, 85, 94).

Universal evidence-based guidelines for anti-infective prophylaxis of CART patients are pending. Although for all patients, herpes simplex (HSV) and varicella zoster (VZV) prophylaxis up to one year after CART treatment and/or until sufficient peripheral CD4 cell counts were reconstituted are recommended (95–97), fungal and bacterial prophylaxes are not routinely recommended after CART treatment. Antibiotic prophylaxis standards vary between different institutions (96, 97), but most regimens include the use of fluoroquinolons (96).

However, in neutropenic CART patients, antibiotic treatment is consensually strongly advised (97, 98), especially considering the high number of infections in these patients (92, 97). A recent study in children reported that infections occurs in about half of them within 3 months before the intervention and in about 40% of the patients in the first days after CART infusion. Bacteria accounted for half of the infections causing a high number of severe and life-threatening bacteremia, notably *E. coli*, *Klebsiella spp*., *Enterococcus spp.* and *Staphylococcus spp* (99). In adult patients, infections were more common within the first 2 months after CART cell therapy, and again bacteria were the most common causative pathogens. Intriguingly, the gut as site of infection and intestinal commensals were found to account for a considerable fraction of infections (89).

## Effects of Antibiotic Treatments on Anti-Cancer Efficacies of Immunotherapies

Antibiotics are commonly used in patients undergoing antitumor therapies to prevent and/or reduce infectious complications. Nonetheless, antibiotics have been shown to promote development of chronic diseases and to affect the clinical outcome of patients treated with immunotherapies (100), which is suggested to be (at least in part) due to negative effects on the gut commensal microbiome. Despite their essential role in managing infections and, thereby, saving lives, there is a growing body of evidence showing that antibiotics have detrimental impact on the antitumor efficacy of T cell-based immunotherapies, notably ICI therapies (see review of the clinical studies in **Table 1**). Such adverse influence on the ICI outcomes are hypothesized to occur through modulation of the intestinal microbiome. Therefore, we will focus in the following chapters on the intestinal microbiome, individual commensals and their potential role in anticancer T cell therapies.

**Table 1:** Outcome of patients treated with immune checkpoint inhibitors (ICI) with or without receiving antibiotic (AB) treatment.

Author	disease	Patients [n]	AB exposure [n]		Clinical response		Overall survival[months or HR [CI 95%]]		Progression-free survival[months or HR [CI 95%]]	
			AB	No AB	with AB	without AB	AB	Non-AB	AB	Non-AB
(101)	NSCLC melanoma/RCC/HCC/H & N/urothelial/others	60	17	43	RR: 29.4 %	RR: 62.8 %	5.5	20.5	HR 1.6 [0.84-3.03]	–
(102)	NSCLC/RCC/urothelial	249	69	180	n.a.	n.a	11.5	20.5	3.5	4.1
(103)	NSCLC	239	48	191	PD: 52 %	PD: 43 %	7.9	24.6	1.9	3.8
	RCC	121	16	105	PD: 75 %	PD: 22 %	17.3	30.6	1.9	7.4
(104)	NSCLC	30	11	19	n.a.	n.a.	7.5	15.1	2.9	3.1
(105)	melanoma	74	10	64	ORR: 0 %	ORR: 34 %	10.7	18.3	2.4	7.3
(106)	NSCLC/melanoma/RCC/H & N	196	29	167	PD: 80 %	PD: 44 %	2	26	n.a.	n.a.
(107)	urothelial	896	235	661	n.a.	n.a.	HR 1.44 [1.19-1.73]	–	HR 1.24 [1.05-1.46]	–
(108)	RCC	69	11	58	RR: 9%	RR: 28%	1.87	5.09	24.6	undefined
(109)	melanoma	568	114	454	n.a.	n.a.	27.4	43.7	n.a.	n.a.
(110)	NSCLC/melanoma/RCC	291	92	199	n.a.	n.a.	10.4	21.7	3.1	6.3

AB, antibiotics; CI, 95% confidence interval; H & N, head and neck; HR, hazard ratio; NSCLC, non-small cell lung cancer; ORR, overall response rate; PD, progressive disease; RCC, Renal cell carcinoma; RR, response rate; N.A., data not available/reported.

## The Intestinal Microbiome as Major Modulator of Mucosal and Immunological Homeostasis

The human body harbors a massive number of microbial members (likewise the number of human cells) that orchestrate a comprehensive range of physiological processes, diseases and cancer susceptibility. Their 100-fold higher gene diversity encodes outstanding mechanism and metabolic competences that influence their own microbial niche, host tissue specific and immune cells function (111). This microbial ecosystem, collectively termed microbiome, is composed of eukaryotes (fungi and protozoa), virus and prokaryotes (112). The majority of commensal bacteria inhabit the colonic gastrointestinal tract while the minority are colonizing other anatomical regions such as oral-respiratory and urogenital tracts, skin as well as tumors. Overall, under healthy conditions, the host and microbiome exist in a symbiotic equilibrium as a metaorganism by providing a nutrient-rich microenvironment in return for aid in digestion and metabolism, respectively (113–115). As such, the microbiome synthesizes vitamins and breaks down food into absorbable nutrients, e.g., carbohydrates, or host signaling molecules such as short-chain fatty acids (116). Differences in geographic location, ethnicity, and dietary habits cause the human microbiome to be highly variable between and within individuals (117). In the last twenty years, research on the microbiome turned to be a field of enormous interest in a broad scientific community, which was leveraged by the Human Microbiome Project 1 and 2. Numerous diseases including cardiovascular diseases (CVDs), inflammatory bowel disease (IBD), diabetes mellitus, cardiometabolic disease, liver disease, neurodevelopmental illnesses, and cancer have been shown to be associated with and even partially driven by alterations of the intestinal microbiome or of microbe-host interactions, termed dysbiosis (115, 118, 119).

Mucosal surfaces of the intestinal tract are constantly exposed to a huge biomass of commensal bacteria, and the host’s response to gut microbes is compartmentalized to the mucosal surface. As primary gate keepers, the intestinal epithelium and a dense layer of mucus separates the lumen with resident microbes from the underlying host’s tissues. The major building blocks in mucus are mucins which are large, highly glycosylated proteins, secreted by intestinal goblet cells. The mucin domain glycans bind a lot of water and thereby generate the typical gel-like properties of the mucus (120). In addition to forming a mucosal gel, goblet cells have been shown to deliver intestinal luminal material to the lamina propria dendritic cells (DCs) for presentation of oral and intestinal antigens to the immune system (121), and also facilitate colonic translocation of commensal bacteria to host lymphatic organs (122). Antimicrobial peptides (AMP), a diverse group of evolutionary conserved defense proteins and peptides, play another critical role in maintaining mucosal barrier functions. Intestinal AMPs are secreted by Paneth cells and, to a lesser extent, enterocytes in the small intestine (123). AMP families include lysozymes (124), cathelicidins (125), α- and β- defensins (126), and regenerating islet-derived (Reg) proteins (127). AMPs mostly exert barrier function through direct bacterial killing, or indirectly via induction of a diverse array of immunomodulatory mechanisms (128). AMPs such as enteric defensins (129), resistin-like molecule β (130), cathelin-related antimicrobial peptide (131) and lectins of the Reg3 family constitutively shape the indigenous commensal repertoire and microbiome ecology (132). In addition, AMPs protect the host from pathogenic infection, e.g. cathelicidin-related peptides against *Salmonella typhimurium* infection (133), while the lectin Reg3γ protects mice against *Listeria monocytogenes* infection (134) and reduces colonization by vancomycin-resistant *Enterococcus* (135).

Secretion of immunoglobulin A (IgA) represents another feature of the intestinal mucosa to protect the host against intestinal pathogens. IgA, the most abundant antibody isotype produced in our body, primarily induced in the Peyer’s patches of the gut, provides non-inflammatory immune protection against *Salmonella typhimurium* (136) or *Enterobacter cloacae* (137). Secretory IgA promotes intestinal immune exclusion by entrapping dietary antigens and microbes in the mucus, down-modulates the expression of proinflammatory bacterial epitopes on commensal bacteria, and, thereby, affects microbial colonization of the gut and maintains a homeostatic ecology of commensal bacteria (138).

These intestinal immunity features are subject to commensal-host mutualisms with extensive bidirectional interaction. For instance, the mucosal expression of Reg3 lectins is regulated by the commensal microbiome through the production of the short-chain fatty acids (SCFAs) propionate and butyrate as major microbial metabolites of Clostridia strains and signaling through G protein-coupled receptor 43 (GPR43) on enterocytes (139). Likewise, the protein and oligosaccharide composition as well as barrier function of the intestinal mucus depends on the colonization with commensal microbes (140). Intestinal secretion of IgA is also determined by the presence of commensal microbes as germ-free animals have ten-fold lower levels of total IgA (141). Specifically, a complex microbiome that contains members of the phylum *Proteobacteria* promotes a T cell dependent induction of systemic IgA that can further protect against polymicrobial sepsis (142).

Besides these non-immunological features of mucosal microbe – host mutualism, the gut microbiome is essential in the ontogeny, maturation and modulation of the adaptive, T cell-driven immunity both in the intestines, extra-intestinal organs and systemically. This interaction starts very early in life because after birth the colonizing gut commensals induce the development of intestinal lymphoid tissues and maturation of myeloid and lymphoid cells, which imprints the immune system with a reactivity level that persists long into adulthood (143). Germ-free mice, in contrast, show defects in multiple specific immunocyte populations such as reduced Th1, Th17 and regulatory T cells in the intestines and mesenteric lymph nodes, impaired cytotoxicity NK cells or compromised innate lymphoid cell (ILC) function (144). In a seminal mouse study aimed to screen for immunomodulatory human gut symbionts, Geva-Zatorsky et al (Cell 2017) reported the effects of 53 individual bacteria on cellular immune phenotypes after mono-colonization of germ-free laboratory mice. Only a handful of symbionts were found to increase T cell frequencies in the intestines, including segmented filamentous bacteria (SFB) and Th17 cells, *Coprobacillus* and IL-10+ T cells, or *Bifidobacterium longum* and IFNγ+ Th1 cells (145). So far, only a few studies have assessed associations between immune cell phenotypes, effector molecules and gut microbiome profiles in healthy humans. For instance, the abundance of the genus *Bacteroides* has been linked to the Th1 cell numbers in the mucosa of the sigmoid colon (146). Furthermore, pathogen-induced immune cytokine responses are hypothesized to be modulated by the intestinal microbiome in healthy humans. *Alistipes, Clostridium* or *Bilophila spp*. were reported to protect against lipopolysaccharide-induced TNFα release from monocytes, whereas *Faecalibacterium spp.* may protect against IL-17 responses (147).

Immune cells recognize and react to small molecules produced by gut commensal microbes such as the above mentioned SCFAs. Along these lines, propionate and butyrate have been identified as major SCFAs that can stimulate the expansion and immune-suppressive properties of the regulatory T cells in the colon either through GPR43 receptor signaling or inhibition of histone deacetylases (HDACs) on the level of DCs (148, 149). Recently, bacterial transformation of bile acids which creates a complex pool of steroids was also observed to induce peripheral regulatory T cells by acting on DCs to diminish their immunostimulatory properties (150). *Bacteroides fragilis* and *thetaiotaomicron* are among the commensals that contribute significantly to bile acid metabolism in the gut (151).

Overall, recent research has greatly enhanced the understandings of the intimate, but complicated crosstalk between the microbiome and the immune system. Nevertheless, many unknowns and challenges remain in disentangling microbiome-immunity interactions in health and disease, notably cancer immunotherapies, which we will cover in the next chapters.

## Gut Microbiome Injuries After Chemotherapy and Irradiation Conditioning for Cancer Therapies and T Cell Transfers

Before widespread use of next-generation sequencing techniques, culture-based methods already provided evidence that chemotherapeutic agents such as 5-fluorouracil (5-FU) perturb the oral and fecal microbiota of laboratory animals with an expansion of gram-negative anaerobes (152). These findings were later expanded by 16S rRNA sequencing results revealing a decrease in *Eubacterium* and *Ruminococcus spp.* as beneficial, SCFA producing bacteria (153). The complex interaction of microbes and chemotherapy is also reflected by pre-clinical results showing that the efficacy and toxicity of the drugs (e.g., 5-FU or irinotecan) depend on the intestinal bacterial composition (154, 155).

In humans, there is only a sparse literature on whether chemotherapy affects diversity and composition of the gut microbiome, and the results are often difficult to interpret due to antibiotic treatments, the development of gastrointestinal toxicities with inflammation and diarrhea, and surgical complications (156–159).

Regarding the toxicity of CART therapies, the most notable toxicities are CRS and neurotoxicity. However, gastrointestinal adverse events were reported in up to 28% of patients in a retrospective analysis by Abu-Sbeih et al. (160). All of them developed diarrhea, but also colitis and bloody stools were observed in rare cases, which can confound any microbiome configuration in these patients. Regarding chemotherapy effects without T cell transfer, in patients receiving a myeloablative conditioning therapy for NHL, chemotherapy was associated with an expansion of *Enterobacteriaceae* and *Enterococcaceae*, and a loss of *Ruminococcaceae, Lachnospiraceae* and *Bifidobacterium spp*. without any additional administration of other drugs such as antibiotics (157). Induction chemotherapy in patients with AML was also observed to reduce the alpha-diversity (i.e., the diversity and species richness within a patient’s biospecimen) of the oral and fecal microbiome during the course of therapy. However, the administration of broad-spectrum antibiotics was found to be the major factor responsible for the loss of diversity in this cohort (156). In patients with advanced colorectal cancer, for instance, adjuvant chemotherapies with irinotecan-, oxaliplatin- and 5-FU-based regimens have also been reported to alter the bacterial and fungal community structure of the gut with outgrowth of *Veillonella, Candida, Malassizia spp.* and loss of *Clostridiales* and *Faecalibacterium spp.* (159). As another anticancer therapy associated with intestinal toxicity, pelvic irradiation for prostate cancer therapy was found to reduce intestinal microbiome diversity, notably in patients developing radiation enteropathy. Radiotherapy also led to a decreased microbial SCFA production capacity and decreased levels of homeostatic rectal mucosa cytokines predisposing to intestinal toxicities and adverse events in these patients (158).

## Effects of the Gut Microbiome On Outcomes Of Allogeneic Hematopoietic Cell Transplantation (allo HCT)

As described above, the intestinal microbiota plays a major role in shaping innate and adaptive immunity (161). Therefore, it is plausible that the efficacy of immunotherapies that rely on peripheral immune cells, such as adoptive cell therapy (ACT) and ICI therapies, may depend on intestinal microbiome configurations and their metabolic outputs as it has been reported repeatedly in recent years (102, 162–164). The role of the microbiome in ICI immunotherapy has already been discussed in extensive reviews (165, 166).

We will rather focus on gut microbiome effects on the outcomes of allogeneic hematopoietic cell transplantation (allo HCT) which is a model and predecessor of modern T cell transfer therapies, such as CART therapy, against hematologic malignancies. During allo HCT, a combination of events such as chemotherapy conditioning, exposure to antibiotics and other drugs such as antacids, or changes in diet greatly affect the structure and function of the gut microbiome leading to injuries and dysbiotic states. As we have shown previously, expansion of *Enterococcus spp*. within the gut microbiome up to a level of mono-domination early after transplant represents a hallmark of dysbiosis in allo HCT patients (167). This expansion was primarily driven by the administration of broad-spectrum antibiotics, but also diets containing specific nutrients such as lactose that nourish enterococci and related facultative pathogens like streptococci and other members of the *Lactobacillales* order. Clinically, an *Enterococcus* mono-domination was associated with reduced overall survival and exacerbated intestinal graft-versus-host disease (GVHD), a major toxicity of allo HCT (167).

Antibiotic treatments and domination events with antibiotic-resistant or pathogenic microbes usually come with a substantial reduction of the diversity of the gut microbiome. Notably, in a multi-centric study with 1362 allo HCT patients, a loss of microbiome diversity early after transplant was associated with a higher risk of transplantation-related death and death attributable to GVHD (168). The risk of relapse, however, was not affected by the diversity. Such a disruption of the intestinal microbial milieu not only leads to the expansion of potential pathogens, but also to a loss of functionality in the host-commensal mutualism. In children undergoing allo HCT and receiving antibiotics, butyrate and propionate, two major microbiome-derived SCFAs, were depleted in the intestinal contents after transplant, and were lower in those children that developed GVHD (169). This is of particular importance as SCFAs are protecting the host, notably gut enterocytes, from deleterious gut GVHD (170).

Besides the development of GVHD, the intestinal microbiome has also been associated with antitumor efficacy and the occurrence of relapse after allo HCT. In a study by Peled et al. (171), a higher peri-transplant abundance of a cluster of intestinal bacteria centered around *Eubacterium limosum* was associated with a decreased risk of relapse/progression of disease. *Eubacterium limosum* is a producer of butyrate, propionate, acetate and lactate (172), and these SCFA haven been attributed to the antitumor efficacy of ICI therapies in humans (163).

The development of relapse after allo HCT is determined by the ability of the engrafting immune system to remove residual leukemia cells through a graft-versus-leukemia (GVL) effect. GVL depends on alloreactive, antigen-specific T cells, NK cell alloreactivity and activated DCs (173). In a large patient-centered study, Schluter et al. (174) described associations of the human gut microbiome with the dynamics of the immune system focusing on peripheral immune cells after allo HCT. Abundances of intestinal *Ruminococcus gnavus* and *Staphylococcus spp.* were positively associated with blood lymphocyte counts and post-transplant reconstitution, whereas *Faecalibacterium* was associated with increases in monocyte rates. Although this study lacks details on the subtypes of lymphocytes and other immune cells, it is the first of its kind to demonstrate a clinical relevance of microbiome-immune interactions in humans which has so far only been reported in animal models.

## Evidence for Connections of the Gut Microbiome With Efficacy and Toxicity of CART Immunotherapy in Hematologic Malignancies

So far, there is only little reporting on the role of the gut microbiome in CART therapy. However, there are several lines of evidence highlighting plausible connections as described above and coming from other preclinical experiments. Intriguingly, the efficacy of ACT against HPV-associated cancers in a mouse model was observed to depend on the microbiome configuration of the host at steady-state by comparing mice obtained from two different vendors (Jackson *vs*. Harlan laboratories) (175). The microbiome differences were attributed to a diverse range of *Bacteroides*, *Prevotella* and *Rikenellaceae*. Following treatment with vancomycin, ACT efficacy was enhanced in Jackson mice, but not in Harlan mice. The increased ACT efficacy was also phenocopied by fecal microbiota transfers, with the microbiome affecting both tumor infiltration and expansion of reactive T cells (175). Along these lines, Paulos et al. reported that translocation of microbiome-derived components such as lipopolysaccharide (LPS) from a radiation-injured gastrointestinal tract activated the innate immune system of tumor-bearing mice and, thereby, enhanced the ACT efficacy through Toll-like receptor (TLR) 4 signaling (176). Antibiotic treatment, in turn, reduced the activation of the innate immune system in irradiated mice, and impaired the effectiveness of ACT. The broad-spectrum antibiotic-induced reduction of ACT efficacy was also shown for adoptive CD4+ T cell transfers against implanted colorectal tumors in mice (177). In the only published data on antibiotic effects in mouse CARTs so far, it was reported that the administration of broad-spectrum antibiotics did not mitigate the tumor killing and survival of mice carrying A20 B-cell lymphomas and treated with CD19-CARTs (177). However, the depletion of the gut microbiome significantly prolonged the persistence of CARTs and the duration of B-cell aplasia in these mice.

The different sensitivity of T cells for ACT and CD19-CARTs to antibiotics may be explained by a differential dependence of these T cells on the intestinal microbiome for executing their effector functions. As described above, irradiation-induced microbial translocation augments the function of adoptively transferred tumor-specific T cells via an increased activation of DCs. As a side note, chemotherapy is also known to cause translocation of bacteria across the intestinal epithelium, and thereby amplifies the function of effector T cells (178, 179). Kuzma et al. also described that the presence of antibiotics in cell culture did not affect T cell expansion and cytokine production during *in vitro* antigenic stimulation (177). The loss of sensitivity to antibiotics by CD19-CART may indicate that microbial translocation occurring during the conditioning process does not impact the function of CD19-CART. This phenomenon may be attributable to the unique feature of CARTs as these cells are equipped to exert effector functions instantly upon tumor encounter without the need to be reactivated by DCs as it is the case in ACT.

Despite the lack of effect of CD19-CART efficacy in mice as shown in this very first study, there is preliminary evidence from human studies pointing towards the role of the gut microbiome and dysbiosis in outcomes of CART immunotherapies. In a single-center study published as meeting abstract, Smith et al. observed differences in the gut microbiome composition assessed by 16S rRNA gene sequencing before CD19-CART infusion associated with therapy outcomes. *Lachnospiraceae* and *Ruminococcaceae* were found to be more prevalent in patients who achieved complete remission, whereas *Peptostreptococcaceae* and *Clostridiales* were more abundant in non-responders; microbiome diversity was not observed to be different in responder *vs*. non-responders (180). Another small study, again published as meeting abstract, reported that the administration of broad-spectrum antibiotics up to 14 days before CART infusion was associated with reduced 3-months response rates. The administration of antibiotics was also found to reduce gut microbiome diversity, and, in turn, facilitated the expansion of enterococci in the patients’ stools (181). These preliminary studies corroborate our hypothesis of associations between microbial diversity and taxonomic composition around the days of CART administration and clinical outcomes of this immunotherapy.

In the following chapters we will focus on mechanistic details of microbe - immune interactions that are of particular relevance for patients receiving CD19-CART immunotherapies, notably the effects of broad-spectrum antibiotic treatments and the role of *Enterococcus spp.* and *Klebsiella spp.* as facultative pathogenic commensals frequently observed in cohorts of blood cancer patients.

## Loss of Function and Other Perturbations of the Gut Microbiome by Antibiotic Treatments

Antibiotic treatment is one of the major causes of perturbation of the human gut microbiome. Alterations in the microbial composition is dependent on dosage, duration of treatment, form of application, and class of antibiotics (182). Various studies investigated the short- and long-term effects on the gut microbiome during and after antibiotic treatment. The most common observation was a decrease of alpha-diversity of the microbiome. For example, a 6-day cefuroxime administration led to a 5% loss of alpha-diversity, whereas 6-days of ciprofloxacin administration resulted in a 40% loss of microbial diversity (183). The loss of diversity was reported to come at the expense of bacteria belonging to *Actinobacteria* and *Firmicutes* whereas, the relative abundance of *Proteobacteria* was found increased (182, 184–186).

Assessing the effects of individual antibiotic classes on the composition of the microbiome, macrolide antibiotics were associated with a decrease of *Actinobacteria*, especially bifidobacteria, and *Firmicutes*, especially lactobacilli, and with an increase of the relative abundance of *Bacteroidetes* and *Proteobacteria* (182, 184). Beta-lactam targeting antibiotics were reported to influence the abundance of *Firmicutes* and *Actinobacteria* negatively while leading to an increase of *Proteobacteria* (184). In addition, cephalosporine administration can also lead to an increase of *Bacteroidetes* (182–184). Glycopeptides do not undergo reabsorption in the gut, and are, thus, considered to perturb the gut microbiome. Vancomycin, for instance, was found to reduce the abundance of *Firmicutes* while increasing *Proteobacteria* abundances, especially *Enterobacteriaceae*, both in human and mice (182–184, 186, 187). Fluoroquinolones, like ciprofloxacin, decreased the abundances of *Firmicutes* and *Actinobacteria*, especially bifidobacteria, but increased the relative abundance of *Bacteroidetes* (182, 184). Clindamycin, a lincosamide, reduced the abundance of *Actinobacteria*, mainly bifidobacteria, and of *Firmicutes*, notably lactobacilli (182). Interestingly, the frequently used antibiotic amoxicillin was found to exert only minor effects on microbial diversity (184, 187).

As presented above on some examples, antibiotic treatments can lead to a loss of potentially beneficial bacteria, resulting in alterations in bacterial metabolites, like SCFA, and colonization with potentially pathogenic bacteria, like *Enterococcus* or *Klebsiella spp*. (188–190).

The relationship between SCFA, including acetic acid, propionic acid and butyric acid, and mucosal homeostasis is well studied (189, 191). SCFAs are involved in several homeostatic processes, including inhibition of histone deacetylases and regulation of hematopoietic cell and non-hematopoietic cell differentiation, resulting in an anti-inflammatory and tolerant condition for the mucosal homeostasis. Furthermore, SCFAs are capable to suppress nuclear factor-κB (NF-κB) in immune cells, resulting in inhibition of the production of proinflammatory cytokines (191–194). SCFAs are also involved in maintaining the physical mucosal barrier as they have been shown to increase transcription of mucin genes and production of mucus in intestinal goblet cells. In addition, cell-cell contacts are influenced by SCFAs, improving tight-junction integrity (191, 195).

Infection of pathogenic bacteria is a major cause of death in cancer patients. To elucidate the mechanisms underlying infections, it is important to take a closer look into the healthy microbiome and its interplay with potentially harmful microbes. There are direct and indirect microbiome-related mechanisms to provide colonization resistance. Direct mechanisms include production of antimicrobial molecules, called bacteriocins, with the ability of killing other bacteria, mostly active against closely related species providing selection advantages. Also known is the ability of microbial metabolites, such as SCFAs, to inhibit growth of pathogenic bacteria, like *Salmonella typhimurium* (189, 196). Indirect mechanisms involve the host’s immune system, which is shaped by the commensal microbiome and involves antimicrobial peptides as described above. The disruption of the gut microbiome by antibiotic treatment has been shown to reduce colonization resistance against pathogenic bacteria with an increased susceptibility to infection (196–198). In laboratory rats, a 14-day ceftriaxone treatment led to increased abundance of *E. coli, Staphylococcus spp*., and hemolytic bacteria that was associated with reduction in fecal propionic acid and higher colonic epithelial permeability and a disturbance of oxidant-antioxidant balance. This mucosal injury was accompanied by increased bacterial translocation, suggesting antibiotic-driven susceptibility to blood-stream invasion of potential pathogenic bacteria (196). In a murine model, a single dose of either streptomycin, clindamycin or a cocktail of metronidazole, neomycin, vancomycin and clindamycin, led to susceptibility to *Listeria monocytogenes* infections. In *ex vivo* experiments, contents of the small intestine of untreated mice, co-cultured under anaerobic conditions, were efficient in eliminating *Listeria monocytogenes*, suggesting a microbiota dependent but immune independent role of colonization resistance. Especially *Clostridia* strains were associated with protection against *Listeria monocytogenes* infection (197). It was also demonstrated that *Bacteroides spp.* effectively inhibited *Salmonella typhimurium* growth by production of propionic acid leading to an intracellular acidification of the pathogen (199). Secondary bile-acids, produced by commensals through 7-α-dihydroxylation of primary bile-acids, as for instance by *Clostridium scindens*, promoted resistance to colonization with *Clostridioides difficile* in mice (200). The role of the gut microbiome in providing resistance against colonization and infection was repeatedly demonstrated in patients with recurrent infection with *Clostridioides difficile*, and restoring a functional microbiome through fecal-microbiota-transfer (FMT) is known to treat recurrent infection more efficiently than antibiotic therapy (201).

Part of the human gut microbiome is the mycobiome, which is barely investigated yet. A recent study showed the influence of antibiotic treatment on fungi in the gut environment. Especially *Candida albicans*, an opportunistic pathogen was found to expand during antibiotic treatment in patients, suggesting a regulatory function of commensal bacteria for *Candida albicans in vivo* growth. Recovery of the microbial community, in turn, led to an effective suppression of the initial outgrowth of the fungus (183). Thus, continuous antibiotic administration may lead to increased susceptibility to intestinal and/or blood-stream infections with *Candida* and other fungi (202).

Colonization and infection with potentially pathogenic bacteria after antibiotic treatment is a widely observed phenomenon, especially in hospitalized patients (186, 189, 190, 197, 199, 201, 203, 204). Underlying mechanisms include the development of antibiotic resistances or the loss of colonization resistance against potential pathogenic bacteria. In addition, intrinsic antibiotic resistances can facilitate colonization and expansion, like resistances of *Enterococcus faecalis* and *Enterococcus faecium* to cephalosporines and aminoglycosides (205).

The development of antibiotic resistances is a major subject of research, even more after it was declared by the WHO as one of the top 10 global public health threats (95). Antibiotic resistance genes (ARGs) are phylogenetically conserved genes and their existence was dated back before the age of antibiotics (206). Due to the excessive use of antibiotics all over the world, the amount of ARGs in commensal and environmental bacteria was presumably never higher as today. It is well documented that the increased ARG-copy number in bacteria correlates with the use of antibiotics (207, 208). As reviewed recently (207), antibiotic pressure drives horizontal gene transfer (HGT) of ARGs with a specific selection of more differentiated ARGs. This selection occurs on the single nucleotide level indicating the high efficiency in gene selection conferring survival advantage. A process that facilitates the development of antibiotic resistance is antibiotic treatment under sub-inhibitory concentrations. It has been shown that antibiotic administration under sub-inhibitory conditions augment gene transfer and gene recombination (208, 209). This effective antibiotic resistance development of potential pathogens is associated with the expansion of individual microbes during antibiotic administration with potentially fatal consequences for the host.

## Effects of Individual Gut Microbes Expanding During Antibiotic Treatment on Immune Regulation and Mucosal Homeostasis

Several microbes are known for their pathologic colonization properties, causing disturbance in the microbial community and contributing to severe infections. Among them are bacteria belonging to the genus *Enterococcus, Klebsiella, Salmonella* and *Streptococcus* (186, 189, 199, 203–205, 210, 211). Various mechanisms of colonization advantages and virulence development were described in recent studies, but little is known about immune regulation during colonization or the infection with these pathobionts.

Clinical and experimental research has focused on the cause and consequence of expansion of *Enterococcus spp.* in hospitalized patient. We have shown previously that antibiotic therapy, but also diet, contribute to mono-domination of the gut with enterococci in allo HCT patients (167), and preliminary data found similar pattern in CD19-CART treated patients (181). As observed in several other clinical conditions, antibiotic treatments facilitate intestinal outgrowth of commensal *Enterococcus spp*., mostly *Enterococcus faecium* and *Enterococcus faecalis*. Recent studies enabled a more comprehensive view on mechanisms forcing a commensal to become a pathogenic bacterium.

*Enterococcus spp.* are extremely flexible in adapting to their environment, as reviewed recently (205), especially in response to environmental stress, e.g., the exposure to antibiotics. A recent study investigated the impact of *Enterococcus faecalis* on the host under various conditions, like mono-colonization or co-colonization with a colitogenic bacterial consortium. Mono-colonization of germ-free mice with two different strains of *Enterococcus faecalis* has been shown to increase the number of DCs and regulatory T cells in the colon at steady-state (145). However, using IL-10 deficient germ-free mice as a background mouse strain that is more susceptible to inflammation, mono-colonization with *Enterococcus faecalis* resulted in a severe inflammation of the colon accompanied by an upregulation of genes involved in stress responses towards unfavorable conditions in enterococci, including protease and chaperone genes and oxidative stress resistance (212). Co-colonization of germ-free, IL-10 deficient mice with *Enterococcus faecalis* together with a colitogenic bacterial consortium, showed an oppositional gene expression pattern involved in growth and replication and only moderate intestinal inflammation. Thus, gene expression and behavior of *Enterococcus faecalis* is dependent on the microbial environment (213). These results indicate a crucial role of the microbial environment in maintaining virulence of enterococci and could explain the ambivalent role of *Enterococcus spp.* as harmless commensal versus being a pathogen. Interestingly, environmental stress in bacteria could also be induced by catecholamines like norepinephrine (214). Norepinephrine led to differently expressed protein patterns, associated with higher bile acid tolerance, aggregation capability and biofilm forming abilities, indicating enhanced environmental resistance with potential role in pathogenesis of colonization and infection during increased stress response of the host (215).

Mechanisms of virulence are, among others, dependent on virulence factors expressed in microbes. Several virulence factors of *Enterococcus spp.* have been shown to interfere with the intestinal environment. Gelatinase E (GelE), a matrix metalloproteinase found in enterococci, is one of the major virulence factors shown to impair the integrity of gut mucosal barriers. *In vitro* experiments showed that the media of GelE-producing enterococci co-incubated with macrophages lead to an altered morphology of intestinal epithelial cells (IECs) (216). *In vivo* mono-associations of germ-free, IL-10 deficient mice with GelE producing *Enterococcus* strains resulted in colitis, whereas colonization with GelE deficient enterococci attenuated the inflammation (212). Other virulence factors associated with aggregation, adhesion or β-hemolysis, contributing to environmental resistance or cell destruction are described in detail elsewhere (217, 218).

Regarding their immune system interactions, *Klebsiella pneumoniae* isolated from various infection sides of hospitalized patients revealed different immune stimulatory patterns in *in vitro* and *in vivo*-experimental studies. Intraperitoneal injection with *Klebsiella* strains, for instance, was associated with lower survival of mice when challenged with strains that induced a low TNFα response in peripheral blood mononuclear cells (PBMCs) *in vitro*, suggesting a potential correlation of immune evasion ability and severity of infection (211).

Amoxicillin treatment in mice led to drastically increased abundance of *Klebsiella variicola* with a significantly increased antibiotic resistance profile and elevated virulence compared to control. Inoculation of antibiotic pre-treated mice with these virulent strains resulted in increased pro-inflammatory cytokine production with more severe colon damage, whereas isolated strains of untreated mice led to almost no inflammation, confirming the hypothesis of antibiotic-enhanced virulence. Inflammation was accompanied by modulated Th1 and regulatory T cell differentiation in peripheral lymphoid tissues. Especially Th1 cells were increased and regulatory T cells were decreased in cervical and mesenterial lymph nodes compared to mice inoculated with *Klebsiella variicola* isolated from non-antibiotic treated mice (219). Besides the effect of antibiotics to induce expansion of individual microbes of the intestinal microbiota, it is hypothesized that antibiotics can favor the *de novo* intestinal colonization with microbes of the oral cavity. Ectopic colonization of germ-free mice with saliva samples of patients with inflammatory bowel disease (IBD) showed a significant *Klebsiella spp.* dependent induction of Th1 cells in the intestinal lamina propria. Other commensals inhabiting the oral cavity failed to induce Th1 cells in these experiments. Knock-out models revealed TLR signaling and IL-18 to contribute to DC mediated Th1 cell induction (220).

Taken together, the expansion of individual microbes can have various impact on the mucosal and systemic immunity. However, there are still several open questions regarding the interplay of individual microbes, their virulence and the host immune system, notably T cell-driven anticancer immunity.

## Potential Mechanisms Underlying Direct Effects of Microbes on T Cells as Models of Microbe – CART Interaction

**Figure 1:**
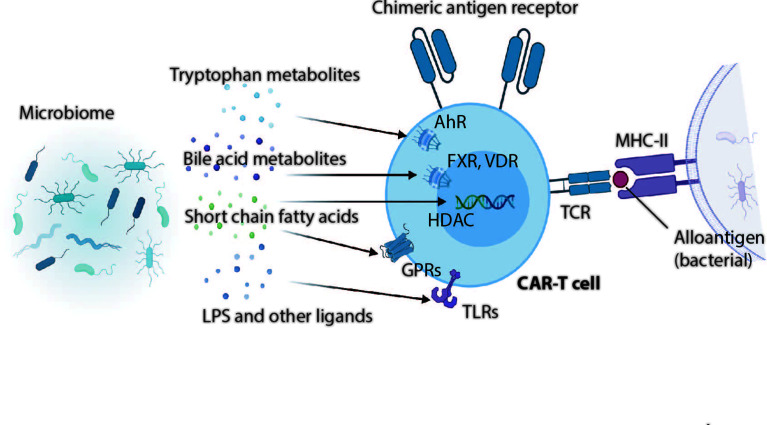
Gut microbial metabolites and microbial ligands can exert far reaching influences on T cells and presumably also CAR-T cells. Whereas tryptophan metabolites can act on T cells through the cytoplasmatic aryl hydrocarbon receptor (AhR), bile acid metabolites induce T cell differentiation and change effector functions through actions on vitamin D receptors (VDR), Farnesoid X receptor (FXR), and also through TGR5, PXR or LXR (no shown in this illustration). Short chain fatty acids act on T cells through G-protein coupled receptors 41, 43 or 109 (GPRs), or regulate immune cell differentiation and function through histone deacetylases (HDACs). Bacterial derived membrane fractions or secreted proteins modulate T cells via Toll-like receptors (TLRs) expressed on activated T cells.

Effector function of T cells defined by the magnitude of cytokine production depends on triggering of the TCR by antigen recognition, engagement of costimulatory molecules and availability of proinflammatory cytokines (221, 222). Increasing evidence indicates that microbial metabolites and cell wall components can regulate the T cell function via host receptors and other target molecules (**Figure 1**).

TLRs are widely expressed in the innate immune system, but certain TLRs such as TLR2, TLR3, TLR5 and TLR9 are also expressed in T cells (223). They can act as costimulatory molecules to enhance proliferation and/or cytokine production of TCR-stimulated T cells. For instance, it has been demonstrated that TLR2 provides co-stimulatory signals to amplify the magnitude of IL-2, TNFα and IFNγ production in murine and human CD8+ T cells, and to increase the percentage of polyfunctional T cells against tumor cells (224). Intriguingly, overexpression of TLR2 in CD19- or mesothelin-targeted CARTs was associated with the expansion, persistency and antitumor function of respective CARTs in mouse models (225). This is of particular interest as commensal enterococci specifically stimulate TLR2 on immune cells via lipoteichoic acid (226); therefore, and based on preliminary data evaluating the impact of the microbiome on toxicity and efficacy of CARTs (181) we hypothesize that an intestinal expansion of enterococci and the co-occurrence of severe CRS in CART treated patients may be due to a direct effect of *Enterococcus spp.* on CARTs via TLR2.

Microbiome derived SCFAs can also act on T cells either through G-protein coupled receptors GPR43 or GPR109 receptor signaling or inhibition of histone deacetylases (HDACs). We have shown recently in an allo HCT mouse model that GPR109 is expressed on activated T cells and significantly contributes to the metabolic fitness of T cells. T cells lacking this receptor are able to proliferate upon antigen stimulation, but undergo activation-induced cell death (227). Notably, antibiotic-induced loss of gut commensal anaerobes leads to a depletion of SCFAs in the host; this, in turn, could explain reduced efficacies of T cell-driven cancer immunotherapies through a reduced metabolic fitness of T cells in an antibiotic-treated host.

By-products of microbial tryptophan metabolism in the gut such as indoles and 5-hydroxytryptophan are further important metabolites that can affect T cell function through the aryl hydrocarbon receptor (AhR). AhR is expressed in different body compartments, but also on T cells. Mounting evidence indicates that AhR plays multiple roles in modulating CD4+ T differentiation and function (228). Recently, it has been shown that 5-hydroxytryptophan induced AhR activation of tumor-infiltrating CD8+ T cells induces a downregulation of cytokines and effector molecules rendering T cells exhausted and dysfunctional in the tumor microenvironment (229). Bile acid metabolites represent another major class of microbiome-derived metabolites that can control T cell differentiation and effector function through the nuclear receptors Farnesoid X receptor (FXR) or vitamin D receptor (150, 230–232).

Besides regulatory and co-stimulatory actions of microbial metabolites on T cell function and antitumor immunity, the concept of “holoimmunity”, i.e., T cell-receptor mediated tolerance against the host and the microbial community residing within the host (233), has emerged as an interesting new area of research in cancer immunity. In this context, cross-reactivity between antigens expressed in commensal bacteria and neoepitopes in melanomas has been demonstrated in a mouse model. In detail, colonization of mice with commensal *Bifidobacterium breve* shape the TCR repertoire to target a bacterial epitope SVY. These T cells cross-react with the model neoantigen SIY on melanomas leading to decreased tumor growth and extended survival in *Bifidobacterium* colonized mice (234). In a recent seminal study, this concept of microbe-associated neoantigens in antitumor immunity was developed even further by demonstrating that bacteria residing within melanoma cells can stimulate an HLA presentation of novel peptides that elicit immune response of tumor-infiltrating T cells (235). Although not extensively studied, a considerable number of bacterial reads has also been recently found in DLBCL tissue (236), but whether bacteria-derived HLA-bound neoantigens also stimulate antitumor T cells or even CARTs remains speculative.

## Approaches to Prevent Dysbiosis or Augment Microbial Homeostasis in the Gut by Microbes, Prebiotics or Diet to Increase the Efficacy of T Cell-Driven Immunotherapies

The problematic role of antibiotics on immunotherapy outcomes has been discussed in previous chapters. If antibiotics, however, are administered to cancer patients because of medical needs such as infections, strategies to protect a healthy microbiome are currently discussed and evaluated. For instance, a colon-targeted antibiotic adsorbent drug has been shown to protect the gut microbiome from moxifloxacin-induced loss of diversity in healthy volunteers (237). This drug is currently investigated in a phase III study with acute myeloid leukemia (AML) or myelodysplastic syndrome (MDS) patients to investigate beneficial effects on the occurrence of life-threatening complications and increased survival.

Another strategy to restore microbial homeostasis after antibiotic-induced microbiome injuries or other dysbiotic states relies on the transfer of a healthy microbiome from a healthy donor to a patient. Such a fecal microbiota transfer (FMT) has been very successfully implemented in clinical medicine as a rescue treatment for *Clostridioides difficile* infections (201). In cancer immunotherapy medicine, two pilot studies recently reported on the induction of *de novo* responses to ICI by FMT in melanoma patients (238, 239).

Apart from transferring whole microbial ecologies, administering individual bacteria as exogenous probiotics has been shown to reward benefits in immunotherapy. For instance, *Bifidobacterium spp*. treatment of tumor-bearing mice improved cancer-specific immunity and response to ICI therapy (240). In other studies, administration of *Bifidobacterium* or *Lactobacillus spp*. was observed to abrogate ICI-associated colitis in mice (241, 242).

As probiotics may be ineffective as exogenous bacteria colonize poorly and live only for a short time in host intestines, prebiotic strategies to enhance endogenous or exogenous microbes in the gut have been developed. Smectite, a type of mineral clay has been shown to promote the expansion of *Lactobacillus* and *Bifidobacterium* by intestinal biofilm formation in mice, and, thereby, enhances the antitumor efficacies of ICI or chemotherapy in tumor mouse models (243).

Diet is considered one of the major modulators of the gut microbiome, and among several nutrients, fibers are essential for microbial homeostasis as they provide essential substrates for microbial growth (244). Low intake of fibers reduces the production of SCFAs and mediates long-term, irreversible shifts in the composition of the microbiome (245). In cancer immunotherapy, a small study (published as meeting abstract) examined the effects of diet and supplement use amongst 46 patients receiving ICIs, and found that patients reporting high-fiber diets were approximately five times more likely to respond to therapy compared to patients with low-fiber intake (246).

To the best of our knowledge, none of the above-mentioned strategies has been studied in CART animal models or within clinical trials. However, these studies would provide enough evidence to initiate trials focusing on dietary or prebiotics approaches to modulate the microbiome and, subsequently, clinical outcomes in CART immunotherapy.

## Conclusion

Multiple clinical and preclinical studies add to the growing evidence that the intestinal microbiome acts in concert with the host in determining antitumor immunity and the outcomes of cancer immunotherapy. For this reason, it is becoming increasingly clear that environmental or external injuries to the microbiome such as administration of broad-spectrum antibiotics can attenuate the efficacy of antitumor immunotherapies that even affect long-term survival. This emerging concept has already led to adjust antibiotic prophylaxis in clinical practice for allo HCT by switching to anaerobe sparing antibiotics (247), or by applying FMT to increase the anticancer efficacy of ICI (239).

**Figure 2:**
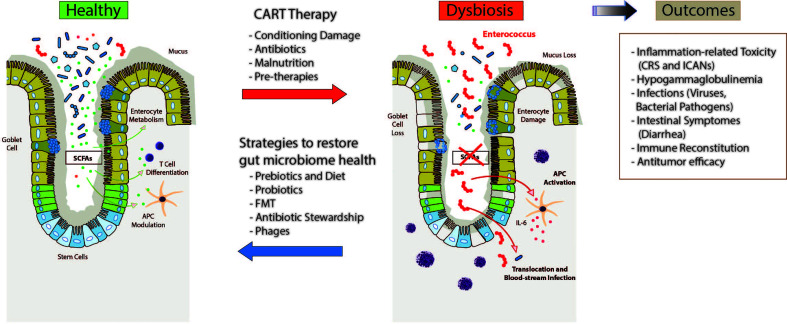
Intestinal microbiome dysbiosis and potential associations with patient outcomes following therapy with chimeric antigen receptor (CAR)-T cells (CART). Patients receiving CARTs are exposed to various environmental conditions including cytotoxic conditioning regimens, antibiotics, and dietary changes or malnutrition that might contribute to changes in the intestinal microbiome. In addition, previous therapies including chemotherapy, stem cell transplantation or even surgeries can affect microbiome homeostasis. These injuries to the intestinal microbiome, in turn, are hypothesized to affect clinical outcome and toxicity to CART treatment, including cytokine release syndrome (CRS), immune effector cell-associated neurotoxicity syndrome (ICANs), infections, gastrointestinal adverse events, immune reconstitution, and relapse through various different immunological mechanisms involving different hematopoietic and non-hematopoietic cell populations. Several strategies have been proposed, although not specifically for CAR-T cell immunotherapy, to restore the intestinal microbiome health which comprise pre- and probiotics, fecal microbiome transfer (FMT), de-escalated antibiotic exposures or even phage-based therapies to mitigate expansion of potential pathobionts.

Due to the short time from their approval, there are only preliminary data suggesting again an important role of the gut microbiome in CD19 CART immunotherapies. In **Figure 2**, we are summarizing plausible perturbations of the microbiome in CART treated patients and their potential impact on the course of the therapy and outcomes. Finally, several modalities are highlighted including dietary interventions through prebiotics, probiotic therapies, FMT and adjustments in antibiotic regimens or phage-based antimicrobial therapies that can help restoring an injured microbiome. Whether these strategies improve response and prognosis of blood cancer patients treated with CART immunotherapies is subject of current studies.

## Author Contributions

M-LS, RR and CS-T wrote the manuscript and performed literature search. MS contributed to concept and interpretation. All authors contributed to the article and approved the submitted version.

## Funding

This work was supported by funding of the Deutsche José Carreras Leukämie-Stiftung to CS-T. M-LS is supported by the Olympia Morata Program of the Medical Faculty of Heidelberg. 

## Conflict of Interest

M-LS: consultancy for Kite/Gilead, Takeda. Advisory board Kite/Gilead, Janssen. None of these sources were involved in the writing of this review. MS: research grants from Apogenix, Hexal and Novartis. Travel grants from Hexal and Kite. Financial support for educational activities and conferences from bluebird bio, Kite and Novartis. Advisory board member of MSD. (Co-)PI of clinical trials of MSD, GSK, Kite and BMS. Co-Founder and shareholder of TolerogenixX Ltd. None of these sources were involved in the writing of this review.

The remaining authors declare that the research was conducted in the absence of any commercial or financial relationships that could be construed as a potential conflict of interest.
